# Breast-conserving surgery following neoadjuvant therapy-a systematic review on surgical outcomes

**DOI:** 10.1007/s10549-017-4598-5

**Published:** 2017-12-06

**Authors:** José H. Volders, Vera L. Negenborn, Pauline E. Spronk, Nicole M. A. Krekel, Linda J. Schoonmade, Sybren Meijer, Isabel T. Rubio, M. Petrousjka van den Tol

**Affiliations:** 1 Department of Surgical Oncology, VU University Medical Center, De Boelelaan 1117, Room 7F-020, 1081 HV Amsterdam, The Netherlands; 2 Department of Plastic, Reconstructive and Hand Surgery, VU University Medical Center, Amsterdam, The Netherlands; 3 Dutch Institute for Clinical Auditing, Leiden, The Netherlands; 4 Medical Library, VU University Amsterdam, Amsterdam, The Netherlands; 5 Breast Cancer Surgical Unit, Breast Cancer Center, Hospital Universitario Vall d’Hebron, Barcelona, Spain

**Keywords:** Neoadjuvant chemotherapy, Breast-conserving therapy, Margins, Cosmetic outcome, Excision volume, Secondary mastectomy

## Abstract

**Purpose:**

Neoadjuvant chemotherapy (NACT) is increasingly used in breast cancer treatment. One of the main goals of NACT is to reduce the extent of local surgery of the breast and axilla. The aim of this study was to determine surgical outcomes for patients receiving breast-conserving therapy (BCT) after NACT, including margin status plus secondary surgeries, excision volumes, and cosmetic outcomes.

**Methods:**

A systematic review was performed in accordance with PRISMA principles. Pubmed, MEDLINE, Embase, and the Cochrane Library were searched for studies investigating the results of BCT following NACT. The main study outcomes were margin status, additional local therapies, excision volumes, and cosmetic outcomes. Non-comparative studies on NACT were also included. Exclusion criteria were studies with less than 25 patients, and studies excluding secondary mastectomy patients.

**Findings:**

Of the 1219 studies screened, 26 studies were deemed eligible for analysis, including data from 5379 patients treated with NACT and 10,110 patients treated without NACT. Included studies showed wide ranges of tumor-involved margins (2–39.8%), secondary surgeries (0–45.4%), and excision volumes (43.2–268 cm^3^) or specimen weight (26.4–233 g) after NACT. Most studies were retrospective, with a high heterogeneity and a high risk of bias. Cosmetic outcomes after NACT were reported in two single-center cohort studies. Both studies showed acceptable cosmetic outcomes.

**Interpretation:**

There is currently insufficient evidence to suggest that NACT improves surgical outcomes of BCT. It is imperative that clinical trials include patient outcome measures in order to allow monitoring and meaningful comparison of treatment outcomes in breast cancer.

## Introduction

Neoadjuvant chemotherapy (NACT) is increasingly used in patients with operable breast cancer. Although these patients do not benefit in terms of survival and local recurrence (LRR) compared to adjuvant chemotherapy, NACT may have several advantages [1–3]. From a surgical point of view, NACT could reduce surgical morbidity of the breast and axilla. By downstaging of the tumor, NACT can convert patients who are candidates for mastectomy to breast-conserving surgery (BCS) candidates [1, 2]. Furthermore, it has potential to reduce excision volumes in patients with large tumors who are already candidates for BCS. Another surgical advantage is downstaging of the axilla so that axillary lymph node dissection can be avoided. Neoadjuvant therapy also permits an early evaluation of the effectiveness of systemic therapy.

In studies involving BCS without NACT, breast conservation is associated with improved cosmetic outcomes and improved aspects of quality of life compared to mastectomy [4–8]. Additionally, smaller excision volumes positively influence cosmetic outcome after BCS [9–14]. Thereby, NACT may also improve cosmetic outcomes through conversion or by lowering resection volumes. However, international guidelines on the use of NACT in lowering breast resection volumes are currently lacking. Multidisciplinary approaches are depending on institutional facilities and expert opinions, resulting in an unaccountable variation between hospitals.

The two main goals of the surgeon when performing BCS are to obtain tumor-free margins and achieve a good cosmetic outcome by keeping the amount of healthy breast tissue excision as low as possible. Tumor-involved margins increase the risk of LRR and therefore require additional local therapy, such as a radiation therapy boost, re-excision, or even mastectomy. These treatments have a negative influence on cosmetic outcomes [9–15]. Unfortunately, the outcomes on margin status are known to be unsatisfactory in a large group of primary BCS patients. For example, a recent nationwide Dutch pathology study showed tumor-involved margins in 16.4% patients after primary BCS [16] and in the United States approximately one out of four patients will undergo one additional surgery after BCS [17]. Over the last decade, one out of three patients were reported to end up with a fair or poor cosmetic outcome [13, 15, 16, 18]. For this reason, surgeons have been investigating the improvement of surgical techniques such as shaved margins, oncoplastic breast surgery, and tumor localization with iodine-125 seeds and ultrasound-guided surgery [19–21].

The same goals as in BCS are being pursued in patients receiving BCS after NACT, although less is known about surgical outcomes. An additional challenge for surgeons performing BCS after NACT is determining the extent and original location of the residual lesion, especially after a good response to NACT.

The initial studies of NACT in breast cancer mainly focused on oncologic outcomes, chemotherapy regimens, molecular subtypes, and the treatment of the axilla. When considering NACT for downstaging of breast tumors, cosmetic outcome is becoming more important, especially since survival and local control is comparable to adjuvant chemotherapy.

## Objectives

The objective of this systematic review was to describe and appraise the literature on surgical outcomes of BCS after NACT compared to adjuvant chemotherapy, including margin status and secondary local therapies, excision volumes, and cosmetic outcomes.

## Methods

### Search strategy and selection criteria

This systematic review was designed and carried out according to the principles of the PRISMA statement for reporting of systematic reviews [22]. A comprehensive search was performed in PubMed, Embase.com, and the Cochrane Library from inception up to May 17th, 2017. Search terms included controlled terms (MesH in PubMed, Emtree in Embase), as well as free text terms. We used free text terms only in The Cochrane Library. Search terms (‘breast cancer OR breast neoplasm’) were used in combination with (neoadjuvant OR induction OR primary systemic) AND (breast-conserving surgery or lumpectomy). Additional keywords (margin, volume, cosmetic outcome, aesthetic outcome) and further logical combinations of these and related terms were used to maximize sensitivity. The reference lists of all identified publications were checked to retrieve other relevant publications.

First, we included studies comparing neoadjuvant with adjuvant chemotherapy in women with operable breast cancer. Because no randomized controlled trials comparing neoadjuvant and adjuvant chemotherapy were found, retrospective and non-comparative studies, were included as well. The search was limited to articles published in English and Dutch. All studies investigating margin status plus the consequent additional local therapies, excision volumes, or cosmetic outcomes were included. No time limit was stipulated. Exclusion criteria were studies with less than 30 patients, studies without marking of the tumor before neoadjuvant therapy, and studies that excluded patients undergoing a secondary mastectomy due to margin involvement.

Two authors (JHV and VLN) independently screened and assessed the records for eligibility and extracted data from the articles. Disagreements on study eligibility were resolved either through consensus or by discussing with a third review author (SM). Whenever necessary, additional data from the authors of the articles were requested.

### Data analysis

The primary outcomes for all studies were margin status with additional local therapies, excision volumes, and/or cosmetic outcomes. Tumor-involved margins were presented as a percentage of all patients undergoing BCS after NACT, while additional local therapies were divided into radiotherapy boost, re-excision, or secondary mastectomy and presented as a percentage of patients undergoing primary BCS after NACT. Excision volumes were reported as means in cc or as mean specimen weights in grams. Cosmetic outcomes could be evaluated subjectively by panel evaluation, by patient self-evaluation or by objective (computerized) measurements, depending on the study design. The Oxford Center for Evidence-Based Medicine (OCEBM) Levels of Evidence was used by both reviewers to help focus on the key concepts for evaluating the internal validity at study level [23]. Meta-analysis could not be performed due to heterogeneity among the studies.

## Results

**Fig. 1:**
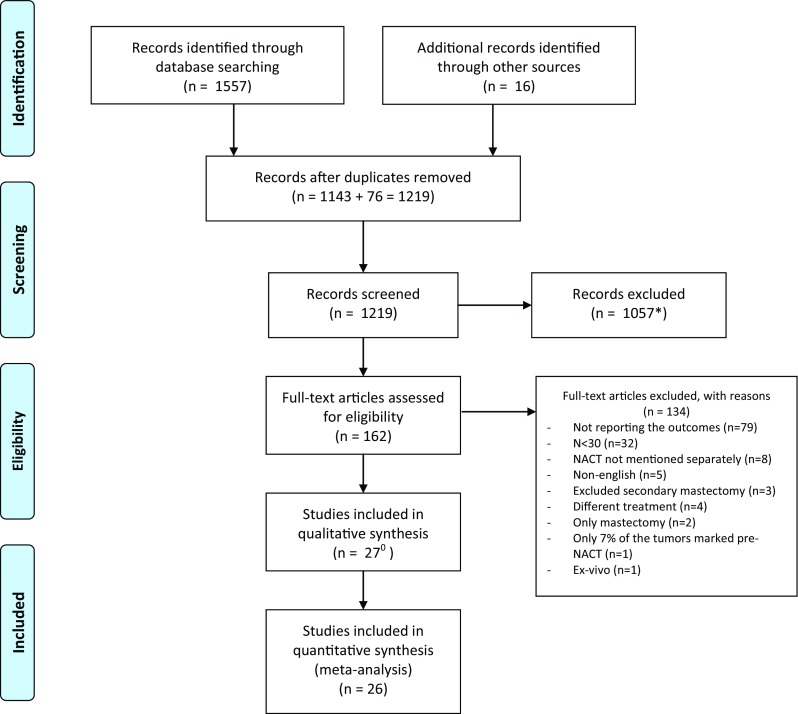
PRISMA 2009 flow diagram. *Including *n* = 90 abstracts eligible for inclusion but are conference abstracts only and were therefore excluded. 0 Articles by Tiezzi et al. [31] and Valejo et al. [55] report on the same group of study participants From Ref. [22]

A total of 1219 unique articles were identified in the database search, after removing duplicates. Sixteen additional manuscripts were included after a manual search through the reference lists of the selected studies. In total, 1057 studies were excluded based on our predefined inclusion criteria after reading the abstracts. One hundred sixty-two articles were fully read and evaluated for reporting of margin status and additional therapy, volume, and/or cosmetic outcome. Eventually, 26 studies were deemed eligible (Fig. 1).

Two meta-analyses described survival and local recurrence rates being comparable between pre- and postoperative chemotherapy [1, 3]. However, none of the studies included in these two meta-analyses reported margin status plus additional local therapies, excision volumes, or cosmetic outcomes.

### Margin status and additional local therapies

In total, 22 studies mentioned margin status plus additional local therapies in patients receiving BCS after NACT [24–45] [Table 1]. All included studies showed a low level of evidence (3 or 4) on the OCEBM scoring system. Ten comparative studies described the surgical outcomes with or without preoperative chemotherapy [24–33]. Positive margins in these comparative studies ranged from 5 to 39.8% after NACT versus 13.1–46% without NACT, leading to secondary surgery in 0–45.4% versus 0–76.5%, respectively. Four retrospective studies reported a significantly lower number of involved margins and secondary surgery after NACT [23–26]. One study, based on a national pathology database describing 626 patients after NACT versus 9275 patients without NACT, reported a higher rate of involved margins (27.3% vs. 16.4%) and secondary surgeries (9.1% vs. 5.3%) after NACT [33]. The other five studies showed no difference between both treatments.

**Table 1:**
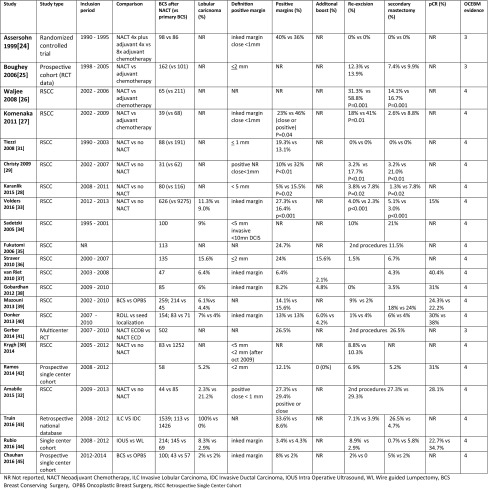
Margin status and additional therapies

Assersohn et al. reported a high rate of involved margins (39.8% vs. 36.4%) in the group receiving four cycles of neoadjuvant plus four cycles adjuvant chemo-endocrine therapy and the group receiving eight cycles of adjuvant chemo-endocrine therapy. All systemic therapy was given concomitantly with radiotherapy and their policy was not to re-resect involved margins. One patient with involved margins had an breast tumor recurrence after a follow-up of 57 months. [24] In a report by Tiezzi et al., re-excision was not performed in all 17 patients (19.3%) with close/involved margins either due to refusal or to technical impossibility (posterior margin involving the major pectoral muscle fascia). A loco-regional recurrence rate of 11% was reported [31].

Twelve non-comparing studies reported 2–33.6% of tumor-involved margins after NACT followed by BCS, with 0–12.4% receiving a re-excision and 0.7–26.5% patients receiving a secondary mastectomy [34–45]. Two studies described secondary surgery after NACT ranging from 11.5 to 26.5%, but this was not further specified as being either a re-excision or a mastectomy [35, 41]. The presence of lobular carcinoma resulted in significantly higher degrees of tumor-involved margins compared with ductal carcinoma.[33, 36, 43] For example, Truin et al. compared lobular carcinomas with ductal carcinomas and reported a secondary mastectomy rate of 26.5% versus 4.7%, respectively [43].

### Excision volumes and cosmetic outcomes

Excision volumes in patients undergoing BCS after NACT were reported in fifteen studies, including two prospective cohort studies and thirteen retrospective studies [20, 25, 27, 28, 31, 33, 39, 40, 42, 44, 45, 47, 48–50]. Thirteen studies were single-center studies. Again, all included studies showed a low level of evidence (3 or 4) on the OCEBM scoring system.

Mean excision volumes and weight ranged from 43.7–268 cm^3^ to 26.4–233 gr, respectively (Table 2). One study was based on data from a prospective randomized controlled trial and compared breast cancer patients receiving BCS before and after chemotherapy. Breast cancer patients with T1–T3, N0–N2 breast carcinoma were randomized between different chemotherapy regimens. The indication for administering pre- or postoperative chemotherapy was not randomized but made by the treating physicians, based on patient and tumor characteristics. In patients with T1 tumors, no significant difference was seen in excision volumes between the pre- and postoperative chemotherapy group, at 98 cm^3^ versus 111 cm^3^, respectively (*p* = 0.51). Tumor size at clinical presentation was 20 mm versus 15 mm (*p* = 0.0055). In patients with T2 or T3 tumors, the mean excision volume was significantly lower in patients who received chemotherapy preoperatively (113 cm^3^ vs. 213 cm^3^, *p* = 0.0043) with no difference in tumor size at presentation [25]. Four retrospective studies that compared BCS with or without NACT reported conflicting results [27, 28, 31, 33]. Two studies showed lower excision volumes after NACT (132.2 cm^3^ vs. 158.1 cm^3^, *p* = 0.04 and 143.6 cm^3^ vs. 273.9 cm^3^, *p* < 0.004) [27, 28], whereas one study showed larger excision volumes following NACT (108 cm^3^ vs. 78 cm^3^, *p* = 0.002) [31]. In a national pathology database study from the Netherlands, no difference was found between the use or absence of NACT in BCS (50 cm^3^ vs. 46 cm^3^, *p* = 0.14) [33].

**Table 2:** Excision volumes

Study	Study type	Inclusion period	Comparison	BCS after NACT (vs. primary BCS)	Tumor diameter (mm)	pCR (%)	Volume measurement	Resection volume or weight	Peroperative localization	OPBS (%)	OCEBM evidence
Boughey 2006 [25]	Prospective cohort (RCT comparing chemotherapy regimens)	1998–2005	NACT versus adjuvant chemotherapy	162 (vs. 101)	Pre-NACT T1 = 20 mm vs. 15 mm (*p* = 0.0055)^a^ T2 = 34.5 mm vs. 30 mm (*p* = 0.143)^a^	NR	(4/3 π (l × w × h ))	T1: 98 vs. 111 cm^3^ (*p* = 0.51) T2: 113 vs. 213 cm^3^ (*p* = 0.0043)	Wire palpation	NR	3
Komenaka 2011 [27]	Retrospective single-center cohort	2002–2009	NACT versus adjuvant chemotherapy	38 (vs. 68)	Pre-NACT 46 mm vs. 33 mm	NR	The product of the 3 diameters	143.6 vs. 273.9 cm^3^ (*p* = 0.003)	NR	NR	4
Tiezzi 2008 [31]	Retrospective single-center cohort	1990–2003	NACT versus no-NACT	88 (vs. 191)	6 mm vs. 19 mm (*p* = 0.01)^b^	NR	1 (4/3 π (l × w × h))	108 vs. 78 cm^3^ (*p* = 0.002)	NR	NR	4
Karanlik 2015 [28]	Retrospective single-center cohort	2008–2011	NACT versus no-NACT	80 (vs. 116)	Pre-NACT 38.4 mm vs. 30.7 mm^a^ Post-NACT 17.3 mm vs. 31.2mm^b^	37%	NR	132.2 vs. 158.1 cm^3^ (*p* = 0.04)	Wire	NR	4
Volders 2016 [33]	Retrospective national database	2012–2013	NACT versus no-NACT	626 (vs. 9276)	NR	17%	(4/3 π (l × w × h ))	50 vs. 46 cm^3^	NR	NR	4
Peintiger 2006 [48]	Retrospective single-center cohort	1987–2002		109	Pre-NACT 35 mm	100%	(4/3 π (l × w × h))	73.12 cm^3^	NR	NR	4
van Riet 2010 [47]	Prospective single-center cohort	2003–2008		47	Pre-NACT 34 mm^a^Post-NACT 8mm^a^	40%	NR	107.25 cm^3^/ 38.61 g	I-125 seed	NR	4
Espinosa 2011 [49]	Retrospective single-center cohort	1999–2009	Tattoo versus marker	149; 118 vs. 31	Pre-NACT 31 mm vs. 32 mm^a^	53% vs. 45%	(4/3 π (diameter^3^)).	268 vs. 143 cm^3^	Palpation Wire Ultrasound Tattoo	NR	4
Donker 2013 [40]	Retrospective single-center cohort	2007–2010	ROLL versus I-125 seed	154; 83 vs. 71	NR	38%	Weight	53 g vs. 48 g	ROLL I-125 seed	NR	4
Mazouni 2013 [39]	Retrospective single-center cohort	2002–2010	BCS versus OPBS	259; 214 vs. 45	NR	24.3% vs. 22.2%	NR	98 versus 180 cm^3^ (*p* < 0.0001)	NR	17.4%	4
Ramos 2014 [42]	Retrospective single-center cohort	2008–2012		58	Pre-NACT 28.3 mm^a^ Post-NACT 11.7 mm^a^	NR	Weight	26.4 gram	IOUS	NR	4
Janssen 2016 [20]	Retrospective single-center cohort	2007–2014		401	NR	37.7%	Formula of a cube	2008: 119.5 cm^3^ 2014: 45.0 cm^3^	I-125 seed	NR	4
Rubio 2016 [44]	Retrospective single-center cohort	2008–2012	IOUS versus Wire	213; 145 vs. 69	Pre-NACT 24.51 mm vs. 24.06 mm^a^	32.4% vs. 43.4%	(4/3 π (l × w × h ))	54.18 vs. 43.72cm^3^	IOUS Wire	NR	4
Chauhan 2016 [45]	Prospective single-center cohort	2012–2014	BCS versus OPBS	100; 43 vs. 57	Pre-NACT 49 vs. 53mm Post-NACT23mm vs 44mm (p0.04)	NR	l × w × h	125.19 vs. 187.54 cm^3^	NR	57%	4
Carrara 2017 [50]	Retrospective single-center cohort	2005–2012		98	Pre-NACT 52 mm	13.30%	Weight	233 g	NR	26,50%	4

*NR* not reported, *NACT* neoadjuvant chemotherapy, *IOUS* intraoperative ultrasound, *BCS* breast-conserving surgery, *OPBS* oncoplastic breast surgery, *ROLL* radio occult lesion localization

^a^Radiological diameter

^b^Pathological diameter

Additionally, ten single-center cohort studies reported excision volumes or specimen weight after NACT without comparing these results with another group. A large heterogeneous patient population was included, and a wide range of excision volumes (43.7–268 cm^3^) and specimen weight (48–233 g) was reported [20, 39, 40, 42, 44, 45, 47, 48–50].

No subanalyses regarding the predictive value of preoperative characteristics, for example, receptor status or histological subtype, and excision volumes were done in any of the fifteen studies. Four studies did analyze the influence of the response to NACT on the amount of volume excised. [33, 42, 44, 48] Volders et al. showed that the median lumpectomy volume in patients with no pathological response was 50 cc, with partial pathological response was 50 cc, and in patients with pathological (near) complete response median lumpectomy volume was 55 cc. (*p* = 0.018). The significant difference seems of low clinical value since there is only 5 cc difference. Moreover, patients with partial and no pathological response had significant more involved margins compared to a pathological (near) complete response (42.1, 25.6, and 12%, respectively). Ramos et al. report no difference in case of complete pathological response (24.4 gr) versus partial response (27.4 gr). [42] Peintiger et al. reported no difference in clinical complete response versus clinical partial response (75.20 cm^3^ vs. 66.78 cm^3^ *p* = 0.53). [48] The volume excised in patients with pCR or minimal pathological residual tumor (Payne–Miller grades 4 and 5) was significantly lower after ultrasound-guided surgery compared to wire-guided surgery. [44].

Mazouni et al. described surgical outcomes of 214 patients after BCS and 45 patients treated with oncoplastic breast surgery (OPBS) after NACT. Excision volumes were smaller in the BCS group (98 cm^3^) compared with OPBS (98 vs. 180 cm^3^, *p* < 0.0001)). In the BCS group, 14.5% were moderately satisfied, 47.9% were satisfied and 37.6% were very satisfied with the cosmetic outcome, which was comparable to the OPBS group (*p* = 0.52) [39]. Karanlik et al. described 251 patients with T2 tumors receiving BCS for invasive breast cancer between 2008 and 2011. Excision volumes were smaller in the patients receiving NACT (158.1 cm^3^ vs. 132.2 cm^3^, *p* = 0.04). Patients’ pictures were evaluated independently by two nurses (median 12 months after surgery) [28]. A good/excellent cosmetic outcome was more common after NACT compared to patients receiving BCS without NACT (92% vs. 80%, *p* = 0.03).

## Discussion

### Margin status and secondary surgery

Based on current data, there is no evidence supporting a positive effect of NACT on tumor-free margins and consequently, on a reduction of secondary surgery. This could be due to a variety of factors. Firstly, an insufficient number of prospective, controlled studies reporting the surgical outcomes have been conducted. Secondly, preoperative imaging and estimation of, non-concentric, residual disease during preoperative imaging appears to be more difficult after tumor downstaging. As a consequence, macroscopic evaluation of the location and extent of residual disease peri-operatively is complicated. It is beyond dispute that marking of the tumor after NACT is essential to achieve identification of residual tumor or the tumor bed and clear margins after BCS. Assessment of current literature indicates that an optimal method for localizing a non-palpable lesion after NACT has not yet been established [38, 40, 42, 44].

The frequency of reported pathological complete response (pCR) after NACT has increased dramatically in the past years due to improvements in targeted therapies, with up to half of all patients in specific groups such as HER2-positive patients [51]. From the studies included in this systematic review, pCR rates range from 12 to 40.4%. [20, 31, 39, 40, 44, 47–50] By definition, these patients will have tumor-free resection margins, and therefore, the estimated percentages of involved margins are actually an underestimate in those patients who have residual disease after NACT. Currently, while the search continues for optimal preoperative imaging to predict response to NACT, the definitive response to chemotherapy is still determined postoperatively by a pathologist.

The importance of achieving tumor-free margins in patients who received BCS after NACT is a matter of debate, especially for patients with a good response to NACT. One of the arguments is the possibility of adjuvant radiotherapy to eradicate microscopic residual tumor and thereby decrease the rate of ipsilateral breast tumor recurrence (IBTR). In the 90s, Assersohn et al. showed a low (1.0%) IBTR after 57 months in 98 patients receiving a ‘sandwich’ schedule of pre- and postoperative chemo-endocrine therapy concomitant with radiotherapy. Breast cancer stages for the patients in the NACT group were not mentioned. Unfortunately, no data are available from prospective trials with current treatment strategies regarding margin width and oncological safety following NACT. In the NSAPB-B18 trial, a significant increase of IBTR was reported in patients who were converted from mastectomy to BCS when compared with those patients who had BCS as initially planned. After 9 years, the rates of IBTR were 15.9% versus 9.9%, a difference that was no longer statistically significant after controlling for patient age and initial clinical tumor sizes [52].

The risk of bias across all included studies is high due to the retrospective character and variation among patient groups, especially in terms of selection and reporting bias. Comparison between studies is not possible and these studies therefore only reflect the current (selected) surgical outcomes after NACT. In general, the group that received preoperative chemotherapy had larger and biologically more aggressive tumors, factors that would be expected to increase the rate of positive margins and re-excision [29]. On the other hand, the studies that did not involve NACT included more lobular carcinomas, which is known to have a higher risk of involved margins.

Nonetheless, the primary goal of every surgeon when starting BCS after NACT is to excise the tumor without tumor-involved margins. Involved margins result in more frequent secondary surgery, leading to poor cosmetic outcomes, additional costs, and psychological stress for the patient. Clinical studies regarding neoadjuvant therapy in breast cancer have mainly been initiated by oncologists, resulting in a degree of neglect of surgical outcomes, a problem illustrated by the lack of data on margin status and excision volumes in randomized controlled trials.

Our view is that both oncological and surgical outcomes after NACT should be assessed equally as the latter positively contributes to a patient’s quality of life, as extensively reported in the adjuvant setting [53, 54]. Furthermore, the distress associated with secondary surgery is also underrecognized in surgical studies. For example, Truin et al. describe 30 secondary mastectomies in 466 patients with lobular cancer undergoing BCS after NACT, concluding that 6.4% of patients undergo a secondary mastectomy [43]. The correct calculation would have included primary lumpectomies only, resulting in a figure of 26.5% (30 secondary mastectomies of 113 primary lumpectomies) (Fig. 1). Patients with invasive lobular carcinoma will have lower pathological complete response (pCR) rates and higher rates of secondary surgery due to positive margins and should therefore be informed about these negative outcomes.

### Excision volume

Neoadjuvant chemotherapy could potentially improve cosmetic outcomes by reducing the volume of excised breast tissue in candidates for both mastectomy and BCS. Among patients with T2 tumors, two studies showed that patients treated with NACT underwent less extensive excision compared with those who underwent primary surgery. Moreover, the smaller resected volumes did not lead to increased risk of re-excision to obtain negative margins [25, 28]. By contrast, Tiezzi et al. reported larger resection volumes in patients after NACT, although it should be noted that these tumors were larger at presentation [31]. It is important to realize that larger volumes do not necessarily result in fewer involved margins or less additional therapy.[16, 25, 27, 47].

With the advent of NACT, the challenge for surgeons became to localize the remaining lesion and to resect the minimum amount of healthy breast tissue, while achieving tumor-free margins.

The pattern of tumor regression is not always concentric and may also occur with diffuse fragmentation. Therefore, the concern of leaving microscopic residual tumor surrounding the surgical area after NACT resulted in a dilemma of how much breast tissue to excise. Plainly, it is not necessary to excise the original tumor volume, otherwise all advantages of performing NACT for downstaging of the breast will vanish [25].

Due to the retrospective and non-comparative design of the currently available single-center cohort studies, no general conclusion can be drawn. The results only reflect the current volumes resected after NACT. Cosmetic failure rates have proven to be significantly higher if the size of the lumpectomy exceeds 40–100 cc, regardless of breast size [9, 12, 13, 18]. Keeping this in mind, almost all of the studies report extremely high excision volumes after NACT, probably resulting in poor cosmetic outcomes for these patients. It would have been of great value if certain subgroups were analyzed for their predictive value on resection volumes, but unfortunately no conclusion can be drawn from the current literature regarding the impact of, for example, receptor status, histological subtype, or chemotherapy regimen on excision volume and cosmetic outcome. The calculated resection ratio (CRR), which is the total resection volume divided by the optimal resection volume, is a measure to assess excessive breast tissue resection. Only one study reported a resection ratio, with a median CRR in the primary surgery and neoadjuvant therapy groups of 3.3 and 2.0, respectively (*p* < 0.0001). This implies that the excision volumes were 2–3.3 times as large as they should be [55].

### Cosmetic outcome

With improvements in NACT strategies, and the increasing rates of pCR with a good prognosis, cosmetic outcomes are becoming increasingly important to breast cancer patients. Unfortunately, any evidence supporting an improvement of cosmetic outcomes after tumor downstaging with NACT is currently lacking. Two single-center retrospective studies reported acceptable results, but included small and highly selected study groups, with a large risk of bias [28, 39]. A poor cosmetic outcome of the breast following breast cancer treatment has a high impact on patients’ quality of life, being a daily reminder of their previous breast cancer and of their treatment period. For this reason, a thorough counseling of patients on the expected primary and secondary outcomes of their treatment is mandatory. In particular, patients receiving NACT to convert from mastectomy to BCS, may have high expectations regarding their cosmetic outcomes and may therefore be unprepared for poor cosmetic results. In particular, when realizing that cosmetic outcomes after a mastectomy with a breast reconstruction may have very satisfactory results as well [7].

One of the main indications for NACT in breast cancer patients to date is tumor downstaging to achieve less morbidity and improved cosmetic outcomes. However, it is interesting that these outcomes of NACT are not supported by any scientific evidence.

The motivation for use of NACT to downstage the breast tumor is based on two facts regarding BCS without NACT:

1. BCS patients have improved cosmetic outcomes and quality of life compared to mastectomy patients [4–8].
2. Excision volume is one of the most important factors determining cosmetic outcome in patients after BCS [9–15, 18].

However, these statements are both based on studies without NACT. Although BCS appears technically feasible in patients receiving NACT after tumor downstaging, a satisfactory cosmetic outcome cannot be guaranteed when still a large amount of breast tissue is resected.

The negative influence of large resection volumes on cosmetic outcomes and the frequent poor cosmetic outcome of BCS without NACT validate current efforts to improve cosmetic outcomes with oncoplastic breast surgery (OPBS). The theoretical advantage of oncoplastic surgery for breast cancer is the possibility of using wider resection margins, leading to improved oncological outcomes and less secondary local therapies, combined with a good cosmetic result. However, involved margins and secondary local therapies after OPBS are still frequently reported [56, 57]. Unfortunately, current literature on OPBS without NACT largely consists of poorly designed and underpowered studies, while high-quality literature on OPBS in patients receiving BCS after NACT is absent. Mazouni et al. reported high rates of involved margins (15.6%) and secondary mastectomies (25%) in OPBS after NACT, while no difference in cosmetic outcome was seen compared to BCS after NACT.[39] Amabile et al. reported secondary mastectomy in 27.3% of patients after NACT followed by OPBS [32].

In conclusion, the advantages of NACT in terms of lower resection volumes and improved cosmetic outcomes after BCT have not yet been proven. Prospective randomized trials including secondary local therapies, resection volumes, and cosmetic outcomes in patients receiving postoperative chemotherapy, as well as reporting the type of initial surgery, will be required to confirm the hypothesis that NACT improves cosmetic outcomes by lowering excision volumes. In a new era of neoadjuvant treatments, it is imperative for surgeons to include not only outcomes of surgical techniques in their routine clinical practice, but also to take note of patient outcome measures in order to better monitor and compare surgical treatment outcomes in meaningful ways.
